# SCAD-Brain: a public database of single cell RNA-seq data in human and mouse brains with Alzheimer's disease

**DOI:** 10.3389/fnagi.2023.1157792

**Published:** 2023-05-12

**Authors:** Xin-Wen Li, Ting-Ting Duan, Jin-Yu Chu, Shi-Yao Pan, Yan Zeng, Fei-Fei Hu

**Affiliations:** ^1^Brain Science and Advanced Technology Institute, School of Medicine, Wuhan University of Science and Technology, Wuhan, Hubei, China; ^2^Community Health Service Center, Geriatric Hospital Affiliated to Wuhan University of Science and Technology, Wuhan, Hubei, China

**Keywords:** Alzheimer's disease, cellular heterogeneity, single cell, RNA-seq, web server

## 1. Introduction

Alzheimer's disease (AD), a severe neurodegenerative disorder, is the most common type of dementia, affecting 57.4 million patients worldwide in 2019 and will reach 152.8 million by 2050 (GBD 2019 Dementia Forecasting Collaborators, 2022). However, there is currently no effective treatment for AD (Gao et al., 2016). Part of the reason is the lacking understanding of the high cellular heterogeneity in AD brains, which is closely associated with the onset, progression, and pathological process of AD (Lau et al., 2020). Single-cell RNA sequencing (scRNA-seq) is extremely effective in interpreting the cellular heterogeneity of AD (Habib et al., 2020). Advances in scRNA-seq technologies in the past decade have generated a large amount of AD scRNA-seq data, but studies integrated these multi-source data are rare, which greatly limited non-bioinformatics users' access to such public data. Therefore, it is essential to establish a database that collects AD scRNA-seq data.

Here, we collected 17 AD or mild cognitive impairment (MCI) scRNA-seq projects from the Gene Expression Omnibus (GEO) and the Synapse databases, covering 21 data-sets with 359 samples, 10 brain regions, 16 major brain cell types, and 1,564,825 cells to explore cellular heterogeneity, cell communication, and cell trajectory in brain tissues with AD/MCI pathology. The results were integrated into a database named SCAD-Brain (scRNA-seq analysis for AD Brain, https://www.bioinform.cn/SCAD/). The users can access, reuse, and analyze the data by visiting the website, to perform cell marker analysis, gene expression analysis, pathway enrichment analysis, cell communication analysis, and cell trajectory analysis for multiple brain regions from patients with AD/MCI and AD mouse models. SCAD-Brain provides a user-friendly platform to explore and visualize scRNA-seq data of AD brain, assist in experiments design, verify hypotheses, and especially investigate the cellular heterogeneity of AD brain.

## 2. Methods

### 2.1. Data collection and quality control

Raw data of AD brain scRNA-seq from the GEO database and Synapse database were collected in October 2021. For data collection through the GEO database, medical subject headings of AD and scRNA-seq were collected from the MeSH database to build a search formula: [(Alzheimer Dementia) OR (Alzheimer Dementias) OR (Dementia, Alzheimer) OR (Alzheimer's Disease) OR (Dementia, Senile) OR (Senile Dementia) OR (Dementia, Alzheimer Type) OR (Alzheimer Type Dementia) OR [Alzheimer Type Dementia (ATD)] OR [Dementia, Alzheimer-Type (ATD)] OR [Dementia, Alzheimer-Type (ATD)] OR(Alzheimer Type Senile Dementia) OR (Primary Senile Degenerative Dementia) OR (Dementia, Primary Senile Degenerative) OR (Alzheimer Sclerosis) OR (Sclerosis, Alzheimer) OR (Alzheimer Syndrome) OR (Alzheimer's Diseases) OR (Alzheimer Diseases) OR (Alzheimers Diseases) OR (Senile Dementia, Alzheimer Type) OR (Acute Confusional Senile Dementia) OR (Senile Dementia, Acute Confusional) OR (Dementia, Presenile) OR (Presenile Dementia) OR (Alzheimer Disease, Late Onset) OR (Late Onset Alzheimer Disease) OR (Alzheimer's Disease, Focal Onset) OR (Focal Onset Alzheimer's Disease) OR [Familial Alzheimer Disease (FAD)] OR [Alzheimer Disease, Familial (FAD)] OR [Familial Alzheimer Diseases (FAD)] OR (Alzheimer Disease, Early Onset) OR (Early Onset Alzheimer Disease) OR (Presenile Alzheimer Dementia) AND [(single cell) OR (single-cell) OR (single-nucleus) OR (single nucleus)], to search datasets on the GEO database. For data collection through the Synapse database, AD scRNA-seq data were collected from the AD Knowledge Portal, which is a repository for multi-omic data on Alzheimer's disease and aging. The information of each sample was manually confirmed according to the metadata and the related published studies. Irrelevant samples (e.g., non-AD or ambiguous disease or AD with genes knocked out) were discarded. After the above procedures, a total of 21 data-sets with 359 samples were collected from public data portals. Raw scRNA-seq data were downloaded from the GEO database and unpacked through the SRA Toolkit (version 2.11.1). The synapseclient and synapseutils python packages were used to download raw scRNA-seq data from the Synapse database.

### 2.2. Contents of data projects

The AD brain scRNA-seq data projects we included in this study are: SRP290191 (Huang et al., 2021), SRP238096 (Sierksma et al., 2020), SRP190819 (Otero-Garcia et al., 2022), SRP254025 (Leng et al., 2021), SRP298630 (Yang et al., 2022), SRP243446 (Habib et al., 2020), SRP252065(Alsema et al., 2020), SRP282056 (Lau et al., 2020), SRP282467 (Xu et al., 2021), SRP124513 (Mathys et al., 2017), SRP305673(Safaiyan et al., 2021), syn18485175 (Mathys et al., 2019), syn24168322, syn16780177, syn12514624, syn21670836, and syn23763501. There are seven mice studies. The SRP290191 and SRP238096 projects explored the response of the microglia system to amyloid-β in pooled cortices (5xFAD, also known as APP/PS1 mice model) and hippocampus (APPswe/PSEN1L166P and THY-Tau22 mice models), respectively. The SRP243446 project focuses on the disease-associated astrocytes population in the 5xFAD mice model. The SRP282467 project elucidated single cell expression of hippocampal tissues from the J20 AD mouse model. The SRP124513 and SRP305673 projects focus on microglia function in AD mice hippocampus (CK-p25, of severe neurodegeneration which develops AD-like pathology in an inducible and temporally predictable manner) and white/gray matter (Trem2KO mouse), respectively. The syn23763501 project performed scRNA-seq for the whole brain of the 5xFAD mouse model. There are ten human studies. The SRP190819 project demonstrates the cell-type-specific responses to neurofibrillary tangles susceptibility in AD prefrontal cortex. The SRP254025 project focuses on selectively vulnerable neurons in AD caudal entorhinal cortex and the superior frontal gyrus. The SRP298630 project concentrates on the vasculature system in the AD hippocampus and superior frontal cortex. The SRP252065 project focuses on microglial subpopulations in AD superior parietal lobe. The SRP282056 project focuses on angiogenic endothelial cells and neuroprotective glia in AD prefrontal cortex. The syn24168322, syn16780177, syn12514624, syn18485175, and syn21670836 projects provide scRNA-seq data from the dorsolateral prefrontal cortex. The 17 data projects were additionally separated into 21 datasets according to species and brain tissues: SRP243446H_Hippocampus_mice, SRP243446C_Prefrontalcortex_mice, syn21670836_DorsolateralPrefrontalCortex_human, syn24168322_DorsolateralPrefrontalCortex_human, syn18485175_DorsolateralPrefrontalCortex_human, syn23763501_WholeBrain_mice, syn16780177_DorsolateralPrefrontalCortex_human, syn12514624_DorsolateralPrefrontalCortex_human, SRP124513_Hippocampus_mice, SRP190819_PrefrontalCortex_human, SRP238096APP_Hippocampus_mice, SRP238096TAU_Hippocampus_mice, SRP252065_SuperiorParietallLobe_human, SRP254025C_CaudalEntorhinalCortex_human, SRP254025G_SuperiorFrontalGyrus_human, SRP282056_PrefrotnalCortex_human, SRP282467_Hippocampus_mice, SRP290191_PooledvCortices_mice, SRP298630H_Hippocampus_human, SRP298630S_superiorFrontalCortex_human, SRP305673_WhiteGreyMatter_mice.

### 2.3. Data processing

For 10x single cell data, CellRanger software (v.6.1.2) (10x Genomics) was used to obtain gene counts by aligning reads to reference genomes (the hg38/GRCh38 for human and mm10/GRCm38 for mouse). Samples with less than 300 cells detected were excluded. For Smart-seq2 single cell data, the Trimmomatic (Bolger et al., 2014) (version 0.39) with default parameters was used to remove adapter sequence and reads trimming, the FastQC (version v0.11.9) was used for quality control (QC), the Hisat2 v2.2.1 (Kim et al., 2019) was employed to align clean reads to mouse reference genome GRCm38, the samtools v0.11.5 (Danecek et al., 2021), and the FeatureCount (Liao et al., 2014) were used to obtain the scRNA expression matrix.

Seurat (Stuart et al., 2019) (version 4.1.0) R package was used to analyze the single cell expression matrix. In detail, quality control of cells was conducted: (1) The percentage of transcripts that map to mitochondrial genes in each cell was calculated; (2) The cells that with >5% mitochondrial genes expressed were discarded; (3) The cells that expressed 200–2,500 genes were remained. Then, cell-level normalization was performed for gene expression by the NormalizeData function in the Seurat R package with default parameters. Highly variable features (HVGs) were identified based on mean gene expression and gene expression variance. Principle component analysis (PCA) and uniform manifold approximation and projection for dimension reduction (UMAP) were accomplished based on HVGs to reduce dimension. After that, clusters of cells were identified by a shared nearest neighbor (SNN) modularity optimization based original Louvain algorithm. Then, cell type clusters were annotated by cross-checking the specifically expressed genes of each cell type, pre-existing cell annotations from published studies, and known cell markers from the CellMarker databases (Zhang et al., 2019).

### 2.4. Statistical analysis

The clusterProfiler (Wu et al., 2021) R package was implemented to conduct the Kyoto Encyclopedia of Genes and Genomes (KEGG) (Kanehisa and Goto, 2000) and Gene Ontology (GO) (Gene Ontology Consortium, 2015) enrichment analysis. The GO enrichment analysis includes three pathway types: cellular component (CC), biology pathway (BP), and molecular function (MF). The Wilcoxon rank sum test was utilized to calculate differentially expressed genes (DEGs) between AD and normal groups. The CellChat (Jin et al., 2021) R package was utilized for cell communication analysis. The Monocle (Qiu et al., 2017) R package was utilized for cell trajectory analysis. Detailed methods are shown on the help page of the SCAD-Brain web site.

### 2.5. Web construction

R/Shiny was applied to construct the user interface and backend of SCAD-Brain. MongoDB database was utilized to store SCAD-Brain data. R packages like DT (Xie et al., 2023), ggplot2 (Wickham, 2016), dplyr, and tidyr were adopted to actualize the data process and data visualization on the web server.

## 3. SCAD-Brain provides services for the reuse and interpretation of scRNA-seq datasets from AD brain tissues

### 3.1. Summary of datasets collected by SCAD-Brain

**Figure 1:**
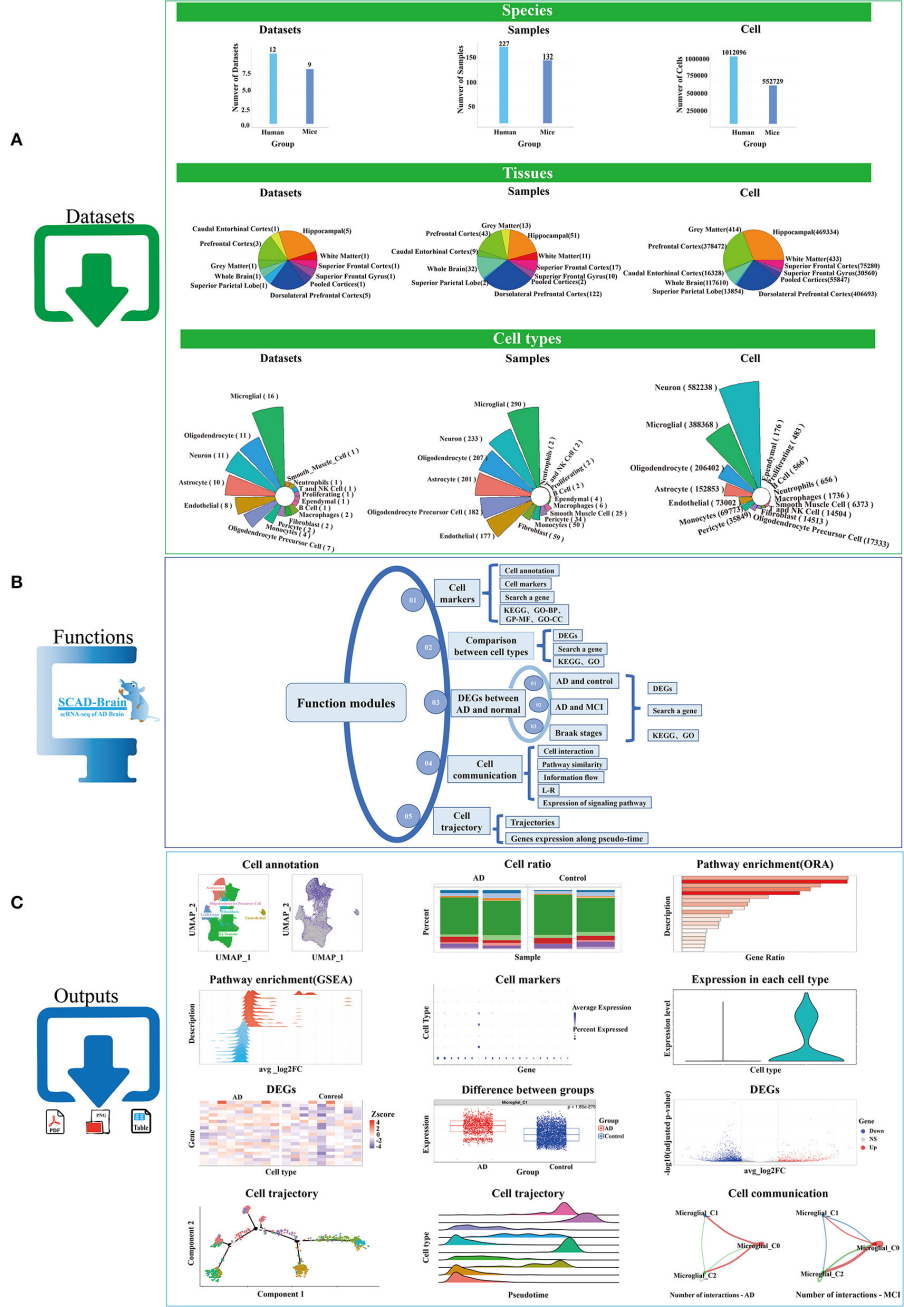
Work flow of SCAD-Brain. **(A)** Statistics of datasets that used in our study. **(B)** Functional modules provided by the SCAD-Brain web site. **(C)** Representative outputs of the SCAD-Brain.

The 17 scRNA-Seq projects we collected cover 21 datasets, two species (human and mouse), 359 samples, 10 brain regions (pooled cortices, hippocampal, prefrontal cortex, caudal entorhinal cortex, superior frontal gyrus, superior frontal cortex, superior parietal lobe, white matter, gray matter, dorsolateral prefrontal cortex), 16 major cell types (oligodendrocyte, astrocyte, microglial, oligodendrocyte precursor cell, endothelial, neuron, T and NK cell, monocyte, B cell, proliferating cell, pericyte, smooth muscle cell, fibroblast, ependymal), and 1,564,825 cells (Figure 1A). Among the 21 datasets (see method part: Contents of data projects), 12 are human studies and nine are mice studies (5xFAD, J20, or APPswe/PSEN1L166P mouse model). Among the 359 samples, 227 are from the human brain and 132 are from mice brains. At present, the hippocampal, dorsolateral prefrontal cortex, and prefrontal cortex are the brain regions that get the most attention in AD research. Additionally, among all scRNA-Seq data in our study, microglial, oligodendrocyte, neuron, and astrocyte cell types have the largest amount of cells.

### 3.2. Functional analyses provided by SCAD-Brain

SCAD-Brain provides a flexible web-based service for the analysis and visualization of collected scRNA-seq data sets from human and mice brains with AD pathology. SCAD-Brain provides four functional analysis types (Figure 1B), including “Cell marker”, “Comparison between cell types”, “DEGs between AD and normal”, “Cell communication”, and “Cell trajectory”. The typical outputs are shown in Figure 1C. Detailed descriptions are shown below.

#### 3.2.1. “Cell markers” module

The “Cell markers” module conducts cell marker investigation and pathway enrichment analysis, including four panels: (1) The “Cell annotation” panel provides cell type annotation for all samples and cell type composition for each sample; (2) The “Cell markers” panel summarizes marker genes for each cell type; (3) The “Search a gene” panel presents the gene expression in each cell type; (4) The “KEGG”, “GO-BP”, “GO-CC”, and “GO-MF” panels provide KEGG and GO pathway enrichment for cell markers.

#### 3.2.2. “Comparison between cell types” module

The “Comparison between cell types” module calculates DEGs between any two cell types, including three panels: (1) The “DEG” panel performs differential expression analysis between two selected cell types; (2) The “Search a gene” panel visualizes the distribution of gene expression in the two selected cell types; (3) The “KEGG” and “GO” panels perform pathway enrichment analysis for DEGs using the Gene Set Enrichment Analysis (GSEA) method, based on the KEGG and GO databases, respectively.

#### 3.2.3. “DEGs between AD and normal” module

The “DEGs between AD and normal” module includes three submodules: “AD and control”, “AD and MCI”, and “Braak stages”. Each submodule has three panels: (1) The “DEGs” panel computes the DEGs between groups; (2) The “Search a gene” panel presents the gene expression in each cell type between groups. (3) The “KEGG” and “GO” panels perform pathway enrichment analysis of DEGs using the GSEA method, based on KEGG and GO databases, respectively.

#### 3.2.4. “Cell communication” module

The “Cell communication” module analyzes ligand-receptor (L-R) interactions among cell types, including five panels: (1) The “Cell interaction” panel summarizes the number and strength of interactions among cell types; (2) The “Functional and structural” panel identifies structurally and functionally similar pathway signals; (3) The “Information flow” panel analyzes the strength of outgoing and incoming signals of each cell type; (4) The “L-R” panel summarizes the communication probabilities of ligand-receptor pairs among cell types; (5) The “Expression of signaling pathway” panel analyzed the expression of genes in enriched signaling pathway between AD and control.

#### 3.2.5. “Cell trajectory” module

The “Cell trajectory” module performs cell differentiation and development trajectory analysis, including two panels: (1) The “Trajectories” panel displays cell trajectories by cell type, cell state, and pseudo-time; (2) The “Gene expression along pseudo-time” panel calculates the gene expression variation according to pseudo-time.

### 3.3. Comparing with the existing AD scRNA-seq databases

As far as we know, there are three databases providing analysis and visualization of scRNA-seq data from AD brain tissues, the TACA (Zhou et al., 2022), the SC2Disease (Zhao et al., 2021), and the scREAD (Jiang et al., 2020). The TACA database was designed to perform various differential expression comparisons to identify cell type-specific gene expression alterations, cell-cell interactions, and drug screening. The SC2Disease database aims to provide a comprehensive and accurate resource of gene expression profiles in various cell types for 22 diseases. However, the data provided by SC2Disease for AD only includes one data set from one brain region (prefrontal cortex). On the other hand, scREAD, a database designed to perform scRNA-seq data analysis for AD that covers multiple brain regions, is currently unavailable due to maintenance issues.

Compared to TACA, SC2Disease, and scREAD, SCAD-Brain covers 10 brain regions and 14 major cell types of AD and adds the following two unique features: (1) comparison between two cell types; (2) cell trajectory analysis. Importantly, although some functions provided by SCAD-Brain are overlapped with these databases, SCAD-Brain provides richer visualization formats. All advancements of SCAD-Brain compared with TACA, SC2Disease, and scREAD are summarized in Table 1.

**Table 1:** Comparisons among SCAD-Brain, SC2Disease, scREAD, and TACA.

	**SCAD-Brain**	**scREAD (lost of maintenance)**	**SC2Disease**	**TACA**
Number of AD related datasets	21	17	1	26
Number of AD brain regions	10	8	1	11
Number of AD brain major cell types	16	7	5	21
Cell Annotation	√	√	√	√
Cell Markers	√	√		
Pathway enrichment of cell markers	√	√		
Comparison between two cell types	√			
Single gene visualization	√			√
DEGs between AD and normal	√	√	√	√
DEGs between stages	√			√
Pathway enrichment of DEGs	√			√
Cell communication analysis	√			√
Trajectory analysis	√			

## 4. Conclusion

We collected and analyzed scRNA-Seq datasets of 10 brain regions from human and mouse models with AD pathology. Additionally, we introduced SCAD-Brain, a database to perform cell marker analysis, GSEA, gene expression comparison between cell types, differential expression analysis between AD and normal or between stages, and cell communication analysis, based on the AD scRNA-seq data we collected. SCAD-Brain is a powerful platform and resource to explore and visualize the cellular heterogeneity of AD brain at single-cell resolution, assist in experimental design and hypothesis testing, and allow users to unravel biological pathways, cell interactions, cell trajectories, and DEGs underlying neurodegeneration in specific cell populations. We will energetically maintain SCAD-Brain and update it with new data and methods.

## Data availability statement

The original contributions presented in the study are included in the article/supplementary material, further inquiries can be directed to the corresponding authors.

## Author contributions

X-WL, T-TD, and J-YC: study design, data collection, web construction, data interpretation and writing for review and editing, and conceptualization. S-YP: data collection and web construction. YZ: study design, writing for review and editing, and conceptualization. F-FH: study design, funding, writing for review and editing, and conceptualization. All authors agree to be accountable for the content of the work and worked collectively to develop the protocols and methods described in this article.
